# *De novo* transcriptomes of cave and surface isopod crustaceans: insights from 11 species across three suborders

**DOI:** 10.1038/s41597-024-03393-y

**Published:** 2024-06-06

**Authors:** Lada Jovović, Jana Bedek, Florian Malard, Helena Bilandžija

**Affiliations:** 1 Ruđer Bošković Institute, 54 Bijenička cesta, Zagreb, 10000 Croatia; 2 Université Claude Bernard Lyon 1, LEHNA UMR 5023, CNRS, ENTPE, F-69622 Villeurbanne, France

**Keywords:** Molecular evolution, Transcriptomics, Molecular ecology

## Abstract

Isopods are a diverse group of crustaceans, that inhabit various environments, including terrestrial, freshwater, and marine, both on the surface and in the underground. The biological mechanisms underlying their wide range of adaptations to diverse ecological niches remain elusive. In order to unravel the molecular basis of their adaptability, we generated a comprehensive RNAseq dataset comprising 11 isopod species belonging to the three different suborders: freshwater Asellota, marine, brackish and freshwater Sphaeromatidea, and terrestrial Oniscidea, with representatives from families Asellidae, Sphaeromatidae, and Trichoniscidae, respectively. Representatives of each family were collected from both cave and surface environments, representing at least three independent cave colonization events. Three biological replicates were sequenced from each species to ensure data robustness. The 11 high-quality RNAseq datasets will serve as a valuable resource for understanding cave-specific adaptations, comparative and functional genomics, ecological annotation as well as aid in conservation efforts of these non-model organisms. Importantly, transcriptomes of eight featured species have been made publicly accessible for the first time.

## Background & Summary

Isopods are a diverse group of terrestrial and aquatic crustaceans with over 10,000 described species, exhibiting a wide range of ecological adaptations^1^. They live in marine, brackish, and freshwater environments, and one lineage also conquered the land. Furthermore, they are one of the most abundant animal groups in caves, with multiple independent colonisations of both aquatic and terrestrial cave habitats throughout the world^2,3^. Understanding the molecular mechanisms that underlie their adaptations to multiple environments is essential for elucidating their ecological success.

Transcriptomics has emerged as an irreplaceable tool across biological disciplines. It is especially important for obtaining molecular sequence information of non-model organisms and provides a foundation for advancing our understanding of genetic diversity, population dynamics, structural variations, selective pressures, and adaptive traits in these species. However, the availability of genomic and transcriptomic resources for isopods is limited, with only a few species having their genomes sequenced or transcriptomes deposited in public databases. Currently, only a few genomes are available, from well-studied terrestrial species, such as *Armadillidium vulgare* (Latreille, 1804)^4^, *Ligia exotica* Roux, 1828^5^ and *Trachelipus rathkii* (Brandt, 1833)^6^, but also a giant deep-sea isopod, *Bathynomus jamesi* Kou, Chen & Li, 2017^7^. Transcriptome data for isopods are more readily available, although isopods represent a diverse group of crustaceans, and the availability of genomic resources varies among different families, genera, and species^8^. For example, just a few species from the families Sphaeromatidae and Trichoniscidae have transcriptomes sequenced but none are cave dwelling^8,9^. Conversely, within Asellidae several RNAseq studies have been published, both on cave and surface representatives of the genus *Proasellus*^10,11^ and the *Asellus aquaticus* (Linnaeus, 1758)^12–15^. Altogether, the genomic data for isopods is limited, especially in comparison to other crustaceans (amphipods and decapods)^16^ or other arthropods like insects.

In this study, we focus on 11 isopod species from three different suborders and families: *Proasellus coxalis* s.l. (Dollfus, 1892), *P. karamani* Remy, 1934, *P. anophtalmus dalmatinus* (Karaman, 1955) and *P. hercegovinensis* (Karaman, 1933) from family Asellidae (Asellota), *Lekanesphaera hookeri* (Leach, 1814), *Monolistra pretneri* Sket, 1964 and *M. radjai* Prevorčrnik & Sket, 2007 from family Sphaeromatidae (Sphaeromatidea), and *Hyloniscus beckeri* Herold, 1939, *Trichoniscus matulici* Verhoeff, 1901, *Alpioniscus balthasari* (Frankenberger, 1937) and *Titanethes albus* (C. Koch, 1841) from family Trichoniscidae (Oniscidea), collected from both cave and surface environments. Utilizing high-throughput RNA sequencing, we generated high-quality *de novo* transcriptomes for each species, with eight of them being sequenced for the first time. This dataset has numerous applications as it enables transcriptomic studies of various isopod species, to explore specific aspects of their biology, adaptation, and ecology. It provides a molecular framework for understanding how isopods from distinct families respond to environmental challenges, as it enables identification of genes and pathways which are involved in cave-specific adaptations. Comparative analyses can reveal both conserved and lineage-specific genes, thus shedding light on the evolutionary history of these crustaceans. Additionally, this dataset can be utilized for ecological annotation of unknown transcripts. The pace at which Next-Generation Sequencing (NGS) datasets are generated is in stark contrast to our current understanding of the functions of the genes they uncover, which remains a significant challenge in genomics and molecular biology. Ecological annotation is an increasingly relevant approach, bridging the gap between gene sequences and their ecological roles. Finally, considering that isopods play a crucial role in many ecosystems, contributing to nutrient cycling and decomposition, fluctuations in their populations can reflect broader ecosystem health. Isopod transcriptomic studies can aid in monitoring and assessing the impacts of environmental disturbances on these ecosystems, and ultimately guide conservation actions.

## Methods

### Work-flow

**Fig. 1:**
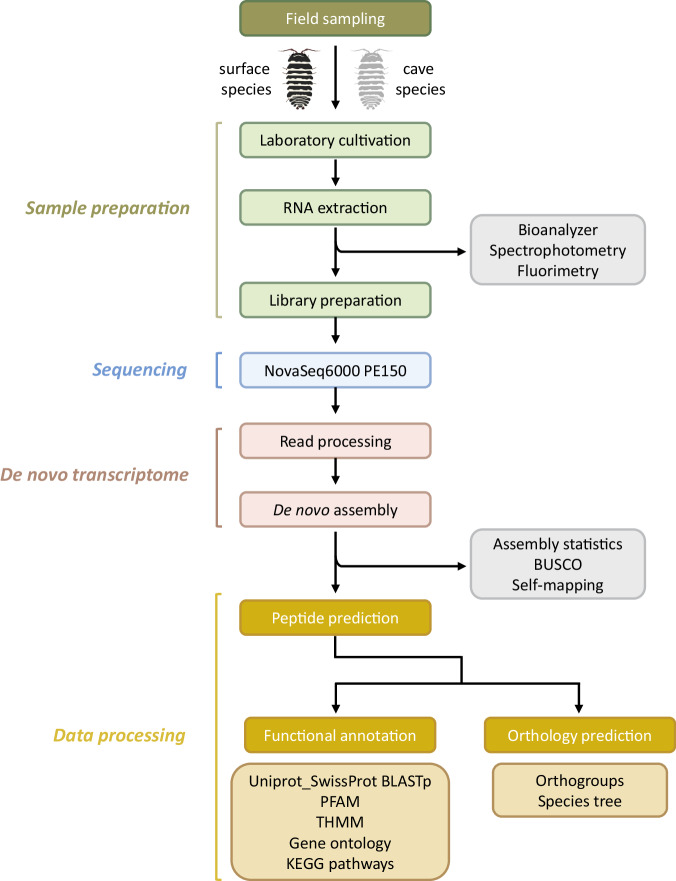
Outline of the experimental workflow and the analysis pipeline.

The overall process, starting with sampling live individuals from nature to *de novo* transcriptome assemblies and all the downstream analysis using various bioinformatics tools and validation steps is summarised in Fig. 1.

### Sample collection

**Fig. 2:**
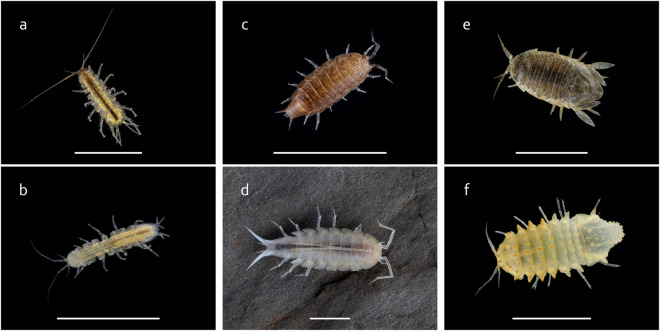
Selected cave and surface representatives of isopod families sequenced in this study. Asellidae (A. *Proasellus karamani*, B. *Proasellus anophtalmus dalmatinus*), Trichoniscidae (C. *Trichoniscus matulici*, D. *Titanethes albus*) and Sphaeromatidae (E. *Lekanesphaera hookeri*, F. *Monolistra pretneri*). Scale bars, 5 mm. Photo credits: Tin Rožman.

Species were selected to obtain independent replicates of subterranean colonization events across three different isopod families. Specimens were collected from cave and surface environments across various geographical locations (Table 1, Fig. 2), using tweezers, brushes, transfer pipettes, large pipettes (turkey basters), nets, and aspirators. Sampling sites for Asellidae were chosen based on a comprehensive search in WAD (World Asellidae Database)^17–19^ database. The selection of localities for Sphaeromatidae and Trichoniscidae was based on an internal database of the Croatian Biospeleological Society. The species were identified according to morphological criteria based on descriptions/redescriptions and identification keys^20–27^. Taxonomic arrangement and nomenclature follow Boyko *et al*.^1^.

**Table 1:** Species used in this study, habitat, sampling location and NCBI Biosample accession numbers of deposited RNA-Seq data.

Family	Species	Abbreviations	NCBI Biosample Accession	Sampling location	Habitat
Asellidae	*Proasellus coxalis* s.l.	PCV_LD	SAMN39098619 SAMN39098620 SAMN39098621	Lateralni kanal Vranskog jezera, Zadar (CRO) 43.94928°N, 15.535606°E	surface/freshwater
	*Proasellus karamani*	PKK	SAMN39098622 SAMN39098623 SAMN39098624	Ključka rijeka, Cerničko polje, Gacko (BiH) 43.0927°N, 18.4852°E	
	*Proasellus anophtalmus dalmatinus*	PAMO_DD	SAMN39098625 SAMN39098626 SAMN39098627	Močiljska špilja, Dubrovnik (CRO) 42.68911°N, 18.07195°E	cave/freshwater
	*Proasellus hercegovinensis*	PHB	SAMN39098628 SAMN39098629 SAMN39098630	Bjelušica, Zavala (BiH) 42.8452°N, 17.9783°E	
Sphaeromatidae	*Laekanespharea hookeri*	LHCR	SAMN39098631 SAMN39098632 SAMN39098633	Crna Rika, Ploče (CRO) 43.05143°N, 17.44922°E	surface/marine, brakish
	*Monolistra pretneri*	MPM4	SAMN39098634 SAMN39098635 SAMN39098636	Špilja kod mlina na Miljacki, Oklaj, Knin (CRO) 44.00348°N, 16,01908°E	cave/freshwater
	*Monolistra radjai*	MRR	SAMN39098637 SAMN39098638 SAMN39098639	Jama u Čapljini, Čapljena, Šibenik (CRO) 43.736655°N, 15.859607°E	cave/brakish
Trichoniscidae	*Trichoniscus matulici*	TPL	SAMN39098640 SAMN39098641 SAMN39098642	Ljuta, Konavle, Dubrovnik (CRO) 42.538463°N, 18.379095°E	surface/terrestrial
	*Hyloniscus beckeri*	HYL	SAMN39098643 SAMN39098644 SAMN39098645	Ljuta, Konavle, Dubrovnik (CRO) 42.538463°N, 18.379095°E	
	*Titanethes albus*	TAT	SAMN39098646 SAMN39098647 SAMN39098648	Tounjčica, Tounj (CRO) 45.248511°N, 15.323145°E	cave/terrestrial
	*Alpioniscus balthasari*	ABM2	SAMN39098649 SAMN39098650 SAMN39098651	Miljacka II, Oklaj, Knin (CRO) 44.000236°N, 16.016247°E	

Coordinates are in the WGS84 format.

Sampled individuals were placed in containers with native water, or, in case of terrestrial species, in a plastic container with a layer of plaster^28^ and transported to the laboratory. Cave species were maintained in complete darkness, whereas surface species were housed in 12:12 hours light:dark cycle. After several weeks or months in laboratory, specimens were randomly selected, and starved for several days. Individuals were flash frozen in liquid nitrogen and stored at −80 °C until further processing. For details on the biology, distribution and ecology of selected species see Lukić *et al*.^28^.

### RNA Extraction and sequencing

Total RNA was extracted from 9 whole individuals per species, in total 99 specimens. SPLIT RNA Extraction Kit (Lexogen, cat # SKU: 008.48) was used for Asellidae and Trichoniscidae. Sphaeromatidae were first homogenized in DNA/RNA Shield (Zymo Research, cat # R1100) and RNA was extracted with *Quick*-DNA/RNA mini prep (Zymo Research, cat. # D7001). The quality of the extracted RNA was rigorously assessed through a combination of several methods: agarose gel electrophoresis and Agilent 2100 Bioanalyzer with RNA 6000 Nano Kit (Agilent Technologies, cat # 5067-1511) for RNA integrity, and spectrophotometry with SPECTROstar Nano Microplate Reader (BMG LABTECH, Germany) for purity. Subsequently, RNA samples underwent TURBO DNAse treatment (Thermo Fisher Scientific, cat #AM2239) until there was no detectable amplification of the marker gene. PCR amplification was performed with GoTaq® G2 Green Master Mix (Promega, cat # M7822,) and the 16 S primers (for Asellidae and Sphaeromatidae 16Sbr-L 5′-CGCCTGTTTATCAAAAACAT-3′ and Stena_R1 5′ -CGTGGAAGTTTAATAGTCGAACAGAC-3′; for Trichoniscidae 16 SarL 5′-CGCCTGTTTATCAAAAACAT-3′ and 16H2 5′-AGATAGAAACCAACCTGG-3′). The thermal cycling protocol consisted of: an initial denaturation at 95 °C for 2 min, followed by 35 cycles of denaturation at 95 °C for 45 seconds, annealing at 52 °C for 45 seconds, and extension at 72 °C for 45 seconds, concluding with a 5-minute final extension at 72 °C. RNA concentrations were measured using a fluorometric approach (QFX Fluorometer and DeNovix RNA Quantification Kit, cat. # KIT-RNA-2-NS, DeNovix). Three individual samples were pooled equimolarly resulting in a total of three biological replicates per each species. Directional or stranded libraries were generated using NEB library prep kits utilizing poly-A selection approach followed by PE150 (paired-end 150 base pairs) sequencing on the NovaSeq. 6000 platform (Illumina) conducted by Novogen Europe, Cambridge, UK.

### Raw reads processing and *de novo* transcriptome assembly

Raw sequencing reads were quality-checked with FastQC^29,30^ and MultiQC^30^ and trimmed for low-quality bases using Trimmomatic^31^. Cleaned reads were rechecked for quality prior to further analysis. If not stated otherwise, all the downstream analyses were conducted on the computer cluster Isabella (University of Zagreb). Species-specific *de novo* transcriptomes were generated from each dataset using Trinity^32^ software.

### Transcriptome quality assessment and mapping

Transcriptomes were analyzed for basic statistics with Transrate^33^ and FastaStatistics^34^. We assessed the completeness of each *de novo* assembly using the Benchmarking Universal Single-Copy Orthologs (BUSCO) software^35^. It involved searching for the presence of eukaryote ‘core’ genes in each assembly, with the Arthropoda database serving as the reference (dataset: arthropoda_odb10 (2020-09-10, 90 genomes, 1,013 BUSCOs).

Self-mapping of reads of each biological replicate against the respective *de novo* assembly, as another measure of quality evaluation, was conducted using Salmon software^36^.

Expression matrices were computed using the Perl script abundance_estimates_to_matrix.pl contained in the Trinity^37^ package for each set of biological replicates separately using Salmon quantification files as inputs. Pairwise Pearson’s correlation coefficients between biological replicates within each species were calculated on TPM matrices in R studio^38^ using cor: Correlation, Variance and Covariance (Matrices) function and visualized with packages reshape2^39^ and ggplot2^40^.

### Protein prediction, functional annotation and orthogroup inference

TransDecoder v5.7.0 (https://github.com/TransDecoder/TransDecoder) was employed to identify open reading frames (ORFs) and predict candidate coding regions, discarding possible non-coding RNA and DNA contamination. Translated transcripts with all types of coding regions (terminal, internal and complete coding sequences) were functionally annotated with ultra-sensitive mode in Diamond^41^ at European Galaxy Serve using BLASTX and BLASTP methods against UniProtKB/Swiss-Prot databases (last update in March 2023). Homology with PFAM common protein domains^42^ was evaluated using profile hidden Markov models or HMMER tool^43^. Transmembrane domains in protein sequences were predicted with TMHMM tool^44–46^ on European Galaxy Server (ExpAA > 80). To elucidate the functional roles of identified transcripts, Gene ontology (GO) and Kyoto Encyclopedia of Genes and Genomes (KEGG) pathway analyses were conducted with EggNOG mapper^47,48^ using web interface (http://eggnog-mapper.embl.de/). Finally, OrthoFinder^49,50^ was employed to analyze protein sequences for phylogenetic orthology inference among 11 isopod species. Species tree has been inferred by using the STAG^51^, rooted using the STRIDE algorithm^52^ and visualized using FigTree v1.4.4 (http://tree.bio.ed.ac.uk/software/figtree/).

## Data Records

The raw full-length reads were deposited in the NCBI *Sequence Read Archive*^53^ with the BioProject accession number PRJNA1056448. For each biosample, two files were submitted, corresponding to one for each of the paired reads. The respective Biosample accession numbers are listed in Table 1. Datasets containing *P. coxalis, P. anophtalmus, P. karamani, P. hercegovinensis, L. hookeri, M. pretneri, M. radjai, T. matulici, H. beckeri, T. albus* and *A. balthasari* transcriptome assemblies were deposited in the NCBI Transcriptome Shotgun Assembly (TSA) database under TSA accession numbers GKUC00000000^54^, GKUB00000000^55^, GKUE00000000^56^, GKUG00000000^57^, GKTY00000000^58^, GKUA00000000^59^, GKTZ00000000^60^, GKUF00000000^61^, GKTX00000000^62^, GKUD00000000^63^ and GKTW00000000^64^, respectively. Datasets containing raw Trinity transcriptome assemblies were deposited in Figshare collection^65^.

## Technical Validation

### RNA and libraries quality control

Only RNA samples with confirmed high quality and integrity (concentration ≥20 ng/μL and flat base line on Bioanalyzer) were used for pooling. It is important to note that RIN number as a measure of RNA integrity can’t be used with isopods since this group, as arthropods in general, shows different numbers of peaks due to presence of hidden breaks in rRNA^66^. Prior to multiplexing, libraries were checked for fragment distribution and concentration to ensure all sequencing criteria have been met (done by Novogene Sequencing company).

### Read and ***de novo*** assembly basic statistics

Sequencing yields, assembly and annotation statistics, completeness and mapping results are shown in Table 2. Briefly, a total of ~180 gigabases (Gb) of raw data were obtained, which is approximately 5.45 Gb per sample on average. The raw paired-end reads ranged between 19,6 to 27,7 million and consisted of a high-quality Q30 score (base error <0.1%) (median Q30 = 93%).

**Table 2:** Summary of RNA seq data generated, *de novo* assembly metrics and annotation statistics.

Family	ASELLIDAE				TRICHONISCIDAE				SPHAEROMATIDAE		
Species	PCV_LD	PKK	PHB	PAMO_DD	TPL	HYL	TAT	ABM2	LHCR	MPM4	MRR
	**Sequence processing**										
**Raw Reads (M)**	22.2	22.8	21.4	21.6	24.0	21.3	21.8	25.9	20.9	24.3	21.8
**Raw bases (G)**	6.7	6.8	6.4	6.5	7.2	6.3	6.5	7.8	6.2	7.3	6.5
**Q30%**	94.3	93.3	93.6	93.6	94.0	92.6	92.0	93.0	92.4	92.9	92.3
**Clean reads (M)**	21.5	22.3	21.0	21.2	23.9	20.8	21.3	25.5	20.4	23.9	21.2
**GC content**	38%	38%	39%	39%	39%	38%	41%	40%	42%	42%	42%
	**Assembly statistics**										
**Transcripts (#)**	207,566	281,607	195,924	152,531	127,009	128,312	152,433	202,875	139,819	149,059	184,458
**Min. length (bp)**	286	282	272	282	282	283	278	278	279	284	280
**Max. length (bp)**	28,657	32,616	22,374	33,782	33,542	34,217	25,314	35,440	28,018	33,050	34,798
**Mean length (bp)**	1,209	1,199	1,094	1,210	1,249	1,195	1,745	1,112	1,264	1,056	927
**Median length (bp)**	574	621	563	600	645	606	592	581	632	515	494
**N70**	1,052	1,022	885	1,070	1,131	1,057	989	918	1,136	831	654
**N50**	2,336	2,051	1,856	2,197	2,185	2,096	1,984	1,893	2,276	1,931	1,437
**N30**	4,075	3,656	3,410	3,790	3,628	3,541	6,003	3,289	3,895	3,451	2,931
**GC (%)**	37%	35%	36%	35%	35%	36%	38%	38%	40.31%	40.44%	40.81%
**L50**	28,518	42,128	28,826	22,324	19,664	19,539	23,869	31,087	20,651	20,788	27,077
**L90**	138,453	190,944	135,633	102,191	84,818	86,498	104,074	140,089	92,917	103,503	134134
	**Transcriptome completeness**										
**Complete (#)**	99.2%	99.3%	98.3%	98.1%	98.5%	99.1%	97.8%	98.0%	98.2%	97.8%	96.7%
**Fragmented (#)**	0.2%	0.4%	1.2%	0.7%	0.7%	0.4%	1.3%	1.0%	1.0%	1.3%	1.4%
**Missing (#)**	0.6%	0.3%	0.5%	1.2%	0.8%	0.5%	0.9%	1.0%	0.8%	0.9%	1.9%
	**Mapping**										
**Overall mapping (%)**	90.70%	90.60%	89.80%	88.80%	93.10%	92.9%	89.60%	90.40%	92.30%	91.90%	88.80%
	**Peptide prediction**										
**Transcripts with coding regions (#)**	104,508	148,300	104,886	69,713	62,239	65,361	71,742	82,123	70,968	72,470	92,023
**Transcripts with coding regions (%)**	50.3%	52.7%	53.5%	45.7%	72.7%	78.8%	41.7%	64.7%	50.8%	48.6%	49.9%
**# complete predicted proteins**	43,858	67,334	41,011	35,805	34,638	33,628	33,147	39,814	34,615	29,604	30,911
**% complete predicted proteins**	42.0%	45.4%	39.1%	51.4%	55.7%	51.4%	46.2%	48.5%	48.8%	40.9%	33.6%
**Min. length (aa)**	86	85	86	85	86	86	86	85	85	85	85
**Max. length (aa)**	9,017	10,289	6,606	9,898	10,399	9,068	8,348	11,334	8,893	10,456	11,136
**Mean length (aa)**	343	354	327	374	426	406	374	377	384	333	287
	**Functional annotation**										
**Coding seq with blastp hits (#)**	55,263	87,548	57,651	38,448	36,584	37,902	38,165	41,447	41,213	40,703	51,459
**Transcripts with blastx hits (#)**	69,456	102,714	67,104	43,446	38,886	40,855	42,196	44,969	43,679	44,948	63,942
**Coding seq with PFAM hit (#)**	63,295	119,143	75,626	43,627	50,065	52,092	42,917	57,298	51,894	45,708	59,778
**Potential TM proteins (#)**	4,376	6,958	4,089	3,260	3,166	3,181	3,033	3,461	3,071	2,364	2,509
**Coding seq with GO term (#)**	39,358	55,685	39,892	27,526	27,177	27,950	28,161	29,730	30,406	29,170	34,620
**Coding seq with KEGG term (#)**	37,164	57,012	37,607	24,662	23,917	24,939	24,789	25,978	27,552	27,830	36,547

Columns are organized by families and species. Abbreviation names for each species are listed in Table 1. Data in *Sequence processing* section represent average across replicates.

**Fig. 3:**
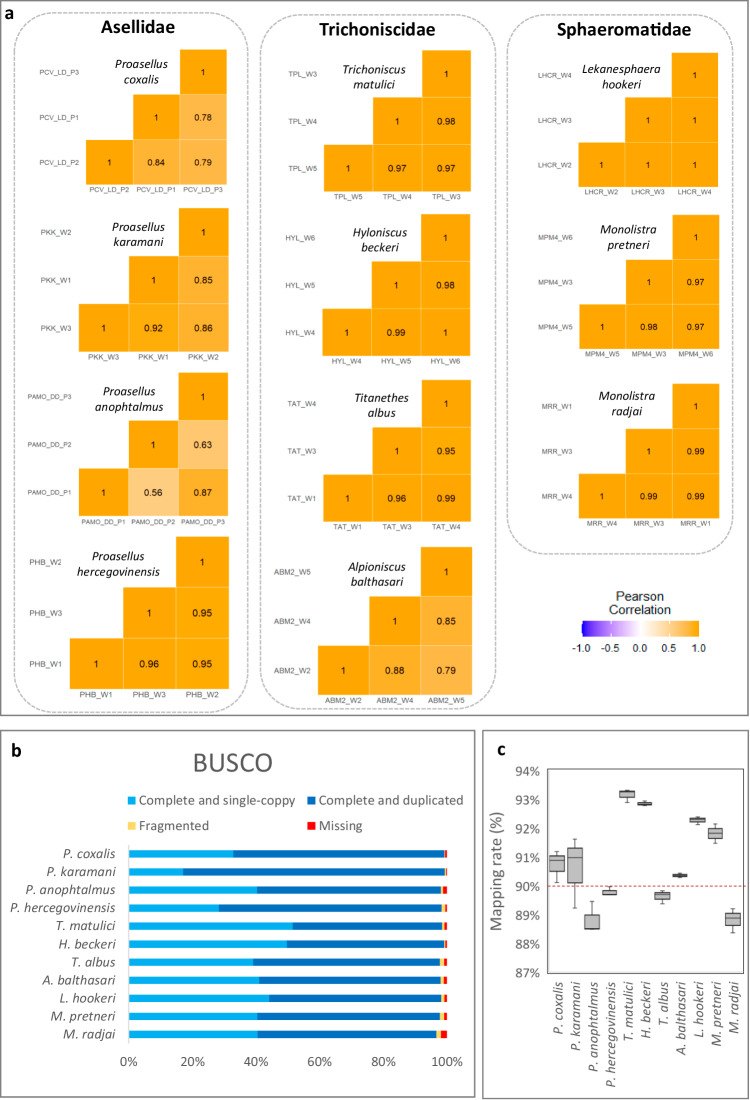
Transcriptome quality assessment. (**a**) Heatmaps of Pearson correlation coefficients between the three replicates for every species. (**b**) Benchmarking Universal Single-Copy Orthologs (BUSCO) scores of the 11 *de novo* transcriptomes. (**c**) Boxplots showing read mapping ratios of RNA-seq data to the *de novo* transcriptomes (three replicates for each species were mapped and the dashed line indicates the average read mapping ratio of the 33 RNA-seq samples).

FASTQC results indicated that cleaned reads passed minimum quality standards. The number of retained reads after filtering and adapter removal exceeded 96% which ensured high level of mapping with an average of 90.81% (88,8% - 93,1%) reads mapped to their respective species-specific transcriptome. Since 80% read mapping is an indication of a reliable assembly according to the standards set by the Trinity protocol, these results demonstrate that our *de novo* assemblies are of high-quality (Fig. 3).

Intraspecies Pearson correlation coefficient between biological replicate pairs was calculated on TPM normalized count matrices (Fig. 3) and it shows good reproducibility and reliability of the experimental design for at least 7 species (Pearson coefficient is 0,95 or higher). Three species (*Proasellus coxalis* s.l., *P. anophtalmus* and *A. balthasari*) have one sample each which shows lower correlation with the rest of the samples (less than 0,80).

The assemblies consisted of 127.009 to 281.607 transcripts (median 152.531). Mean contig length is ranging from 927 to 1745 and contig L50 from 19,539 to 42.128. All transcriptomes had N50 values that exceeded 1,000 bp. The smallest transcript was 272 bp long (in *P. hercegovinensis)*. The longest transcript across all eleven assemblies was between 22.374 and 35.440 bp long.

Busco analysis revealed that transcriptomes are of high quality with 96,7% of “complete” orthologous BUSCO “core” genes being the lowest score among all samples (median 98,2%) (Fig. 3).

Overall results indicate that sequencing has yielded in high-quality RNAseq datasets for 11 isopod species.

### Protein prediction and annotation

At least 51% of the transcripts had ORFs with maximum of 78,8% of all transcripts with coding regions in *Hyloniscus beckeri* transcriptome. Predicted proteins had on average minimum lenght of 85 aa (amino acids) and mean lenght of 362 aa (with maximum of 11.334 aa in *A. balthasari*). Out of these predicted proteins, on average 46% ORFs were complete and include START and STOP codon.

Annotation analysis revealed that the percentage of coding sequences with significant BlastP hits was similar across all eleven assemblies, with at least 50% in UniProt/SwissProt database, 36% in GO and 32% of sequences being annotated in KEGG. Moreover, at least 60% had a recognizable protein domain and on average 4% of coding sequences had predicted transmembrane helices which makes them potential integral membrane proteins (Table 2).

### Orthology prediction and phylogenetic relationship confirmation

**Fig. 4:**
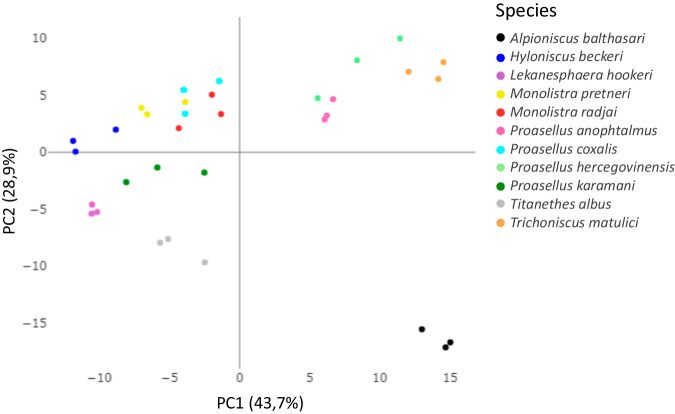
Principal component analysis (PCA) of the expression levels of 15 single-copy orthologues among 11 isopod species. The proportion of variance explained by each principal component is provided in parentheses along each axis.

Orthofinder^49,50^ was utilized to detect potential orthologs and group proteins into orthogroups for the eleven species (Table 3). Only the longest predicted protein by Transdecoder was used in the analysis. A total of 848.798 predicted proteins (89,9%) were assigned to 85.288 orthogroups. 8,673 orthogroups (10.2%) were found to be shared among all 11 species. 100,771 predicted proteins were identified as species-specific, and were categorized into 24,687 inferred orthogroups. Principal component analysis (PCA) and hierarchical clustering of the TPM expression levels of 15 single-copy orthologues among all isopod species revealed that samples primarily clustered according to the species with first two components explaining more than 72% of the variance (Fig. 4).

**Table 3:** OrthoFinder statistics.

Elements	Value
Number of species	11
Number of genes	944,333
Number of genes in orthogroups	848,798
Number of unassigned genes	95,535
Percentage of genes in orthogroups	90%
Percentage of unassigned genes	10%
Number of orthogroups	85,288
Number of species-specific orthogroups	24,687
Number of genes in species-specific orthogroups	100,771
Percentage of genes in species-specific orthogroups	11%
Mean orthogroup size	10
Median orthogroup size	4
G50 (assigned genes)	25
G50 (all genes)	21
O50 (assigned genes)	8,978
O50 (all genes)	11,046
Number of orthogroups with all species present	8,673
Number of single-copy orthogroups	15

**Fig. 5:**
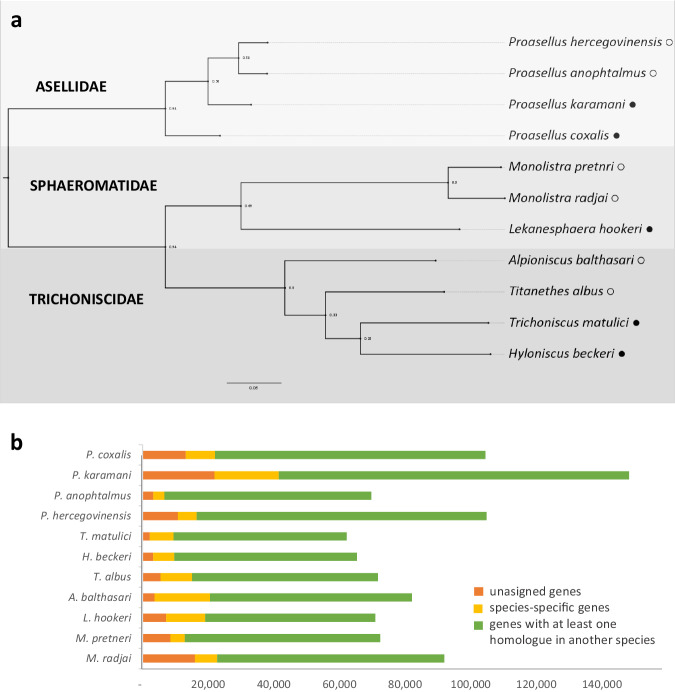
Orthology analysis across the sampled isopod species. (**a**) The consensus species tree based on gene trees of 8.673 orthologues present in all species (the support value for each bipartition is the number of individual species trees that contained that bipartition; black and white circle represents surface and cave species, respectively). (**b**) The number of coding sequences which are unassigned to any orthogroup (orange), species-specific (yellow) or shared in at least another species (green).

Orthofinder also generated a species tree based on 8.673 orthogroups with orthologues present in all species^51,52^. In the ortholog phylogram (Fig. 5), a distinct separation among three suborders (Asellota, Oniscidea and Sphaeromatidae) is evident, aligning with expectations^67^. Species from the *Monolistra* and *Proasellus* genera constitute a monophyletic group. Cave representatives are clearly distinct in all three suborders. There are no molecular family-level phylogenies of Trichoniscidae and Sphaeromatidae, but recent molecular phylogeny studies support our phylogenetic reconstructions for the family Asellidae^19^. It is important to note that this phylogram was not constructed based on an evolutionary model but solely relies on the substitution rates of single-copy orthologs.

## Data Availability

Software tools used, with versions and any parameters differing from default are described below: **1. FastQC** v0.11.8^29^ **2. MultiQC** v1.13^30^ **3. Trimmomatic** v0.39^31^, parameters: SLIDINGWINDOW:4:20 LEADING:5 TRAILING:5 MINLEN:25 **4. Trinity** v2.8.6^32^, parameters: -seqType fq -min_contig_length 300 -min_kmer_cov 1 -min_glue 2 -KMER_SIZE 25 -SS_lib_type RF -CPU 8 -max_memory 80 G **5. Bowtie2** v2.4.4^68^ **6. Jellyfish** v2.3.0^69^ **7. Salmon** v0.14.1^36^ **8. Samtools** v1.9^70^ **9. FastaStatistics**^34^ on Eruropean Galaxy server^71^ **10. TransRate** v1.0.3^33^ **11. BUSCO** v5.4.3^35^, parameters: dataset arthropoda_odb10 (Creation date: 2020-09-10, number of genomes: 90, number of BUSCOs: 1013 **12. TransDecoder** v5.7.0, parameters: default (open reading frame >100 amino acid) **13. Diamond** v2.0.15^41^ on European Galaxy server^59^, parameters: -s ggnog_swissprot_2023_03 -query-gencode ‘Standard Code’ -strand ‘both’ -comp-based-stats ‘Default mode, Hauser 2016’ -min-orf 1 -ultra-sensitive -algo ‘Double indexed (0)’ -matrix ‘BLOSUM62’-comp-based-stats ‘1’ -masking ‘Tantan’ -max-target-seqs ‘1’ -evalue ‘1e-05’ -id ‘0’ -query-cover ‘0’ -subject-cover ‘0’ -block-size ‘0.4’ -motif-masking ‘Disabled (0)’ **14. Hmmer** v3.3.2^43^ **15. UniProt**: (http://www.uniprot.org/help/uniprotkb), March 2023 **16. TMHMM** v2.0^44–46^ **17. eggNOG-mapper** v2.1.12^47,48^, parameters: -m diamond -evalue 0.001 -score 60 -pident 40 -query_cover 20 -subject_cover 20 -itype proteins -tax_scope auto -target_orthologs all -go_evidence all -pfam_realign none -report_orthologs -decorate_gff yes -excel **18. Orthofinder** v2.5.4^49–52^, parameters: -S diamond **19. FigTree** v1.4.4 (http://tree.bio.ed.ac.uk/software/figtree/)
